# Work-related airway symptoms, nasal reactivity and health-related quality of life in female hairdressers: a follow-up study during exposure

**DOI:** 10.1007/s00420-012-0835-8

**Published:** 2012-12-23

**Authors:** K. Kronholm Diab, B. A. G. Jönsson, A. Axmon, J. Nielsen

**Affiliations:** Occupational and Environmental Medicine, Department of Laboratory Medicine, Lund University, 221 85 Lund, Sweden

**Keywords:** Bleaching powder, Work-related nasal symptoms, Nasal lavage, Diary, Health-related quality of life

## Abstract

**Objectives:**

Hairdressers often complain of work-related rhinitis (WR). They are infrequently sensitized to persulphates. The cause and mechanism of the symptoms and the effects on their health-related quality of life (HRQoL) remains unclear. The objectives were to follow female hairdressers with WR mainly from bleaching powder regarding nasal reactivity to persulphate and to evaluate symptoms, HRQoL and inflammatory markers in nasal lavage during a working period after vacation and compared with hairdressers without symptoms and pollen allergic women.

**Methods:**

Skin prick tests to persulphate were performed in the hairdressers. Participants kept a diary of symptoms and of work tasks (hairdressers only). They completed HRQoL questionnaires. Eosinophil cationic protein (ECP) in nasal lavage fluid was examined. The symptomatic hairdressers performed nasal challenges with persulphate before and after the exposure.

**Results:**

Skin prick tests were negative. Although the nasal reactivity to persulphate did not change a steady increase in nasal symptoms, especially blockage, and in ECP was noticed in the symptomatic hairdressers. The HRQoL deteriorated in the symptomatic hairdressers indicating an effect on their working situation and daily life. The atopics had more, but varying symptoms (itching, sneezing and secretion).

**Conclusions:**

The difference in the clinical picture between the symptomatic hairdressers and the pollen allergic women, the increase in symptoms and ECP in the nasal lavage support the view that a sensitization to hairdresser chemicals by a mechanism not yet understood is operating. The deterioration of the HRQoL in the symptomatic hairdressers indicates a considerable effect on their life.

## Introduction

Hairdressers often complain of work-related airway symptoms. They are exposed to several irritating and sensitizing agents, but they often relate their symptoms to bleaching powder (Albin et al. 2002; Brisman et al. 2003). Persulphates found in bleaching powder have often been blamed because they are irritating and sensitizing agents causing both rhinitis and asthmatic symptoms. Specific challenge to persulphate has been suggested as an useful tool in diagnosis of occupational asthma in hairdressers (Muñoz et al. 2004). However, specific IgE antibodies against persulphates are seldom found (Parra et al. 1992) and another immunologic mechanism not yet elucidated has been suggested (Moscato et al. 2005; Muñoz et al. 2004). Furthermore, the clinical picture is quite complex as hairdressers reacting to bleaching powder very often complain of symptoms associated with exposure to other hairdressers chemicals. In a previous study, we found that hairdressers with nasal symptoms from bleaching powder reacted to a nasal challenge with potassium persulphate in the same way as atopics without earlier exposure to bleaching powder (Kronholm Diab et al. 2009). This reaction was associated with a Th1 cell activation, which may be a part of the process of hyper reactivity from low irritant exposure (Banauch et al. 2005; Van Loveren et al. 1996).

In an earlier study (Kronholm Diab 2002), hairdressers claimed that their work-related symptoms increased during periods of exposure and also that they became more sensitive to other stimuli as well, indicating an increasing reactivity in the nasal mucosa. They felt that the reactivity decreased considerably during time away from work. For this reason, frequent periods without exposure were necessary for the hairdressers to be able to continue work.

Health-related quality of life (HRQoL) has been introduced late in occupational medical research compared to care health research in general. HRQoL and working life are linked and must be of concern to occupational health researchers (Blanc 2004). Data indicate that allergic rhinitis may have an important impact on productivity because of symptoms as tiredness, poor concentration and headache (Blanc et al. 2001).

The mechanisms of hairdressers’ nasal symptoms and the consequences for their HRQoL are not clear. This is problematic when hairdressers ask for medical advice concerning continued work as a hairdresser. To clarify this issue, further research about the symptom mechanism and the influence of the symptoms on HRQoL during exposure periods is of great need.

The objectives of this study were to follow the development of work-related airway symptoms, HRQoL and inflammatory markers in nasal lavage fluid during a working period after vacation in female hairdressers. Hairdressers with mainly bleaching powder–related nasal symptoms were compared with hairdressers without such symptoms and pollen allergic females during the pollen season. Furthermore, we studied changes in nasal reactivity to persulphate in the symptomatic hairdressers.

## Materials and methods

### Study design

**Fig. 1:**
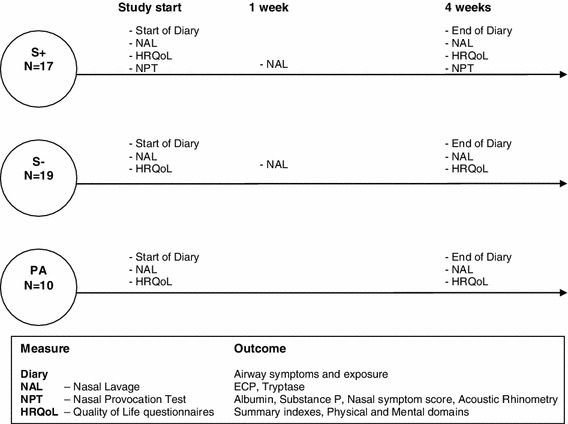
Flow chart of the study design, the measures and the outcomes (*S*+ symptomatic hairdressers, *S*− asymptomatic hairdressers, *PA* pollen allergic women)

The study is a short-term prospective study of hairdressers with work-related nasal symptoms from bleaching powder using a diary of symptoms and work tasks during 4 weeks after at least 2 weeks off work. As controls, one group of asymptomatic hairdressers and another one of females with hay fever to pollen were recruited. At the beginning and at the end of the study, the participants filled in HRQoL questionnaires and nasal lavage fluid was obtained for analyzing of albumin, eosinophil cationic protein (ECP), tryptase (for the atopic group) and Substance P. Another nasal lavage was performed in the hairdressers after a week of work. A medical examination was carried out in all participants before study start. The symptomatic hairdressers also performed a specific nasal challenge with potassium persulphate before and after the exposure period. Figure 1 shows the measures in each group and the outcomes.

The study was approved by the Regional Ethical Review Board at Lund University. All subjects were informed of the purpose of the trial and gave their signed informed consent.

### Study population

We recruited symptomatic hairdressers among patients from the department of Occupational and Environmental Medicine in Lund and through a systematic telephone campaign to hairdresser salons in southern Sweden. The asymptomatic hairdressers were recruited in the same campaign. The inclusion criteria for the target group of hairdressers (Group S+; *n* = 17) were clear nasal symptoms to bleaching, but they could have symptoms from other hair chemicals as well. The latency time until nasal symptoms was 5 years (1–34) (median; range). In three persons, it was not possible to define a latency time. The comparison group were without symptoms (S−; *n* = 19). Exclusion criteria for all groups were history of atopy and/or asthma. Pollen allergic women were recruited among former research participants and by contact with the Department of Otorhinolaryngology, the division for Allergy, Lund. We included the pollen allergic group because of the well-known mechanism of symptoms, and the established impact on their quality of life. The pollen allergic group (PA; *n* = 10) had nasal symptoms only from birch and/or grass and Chrysanthemum Weed, no exposure to bleaching powder and took no regular medications for allergies. Difficulties to find women with allergy to merely pollen made this group smaller than the hairdresser groups.

The content of pollen/m^3^ air and day (alder, hazel, birch, grass and Chrysanthemum Weed) varied between low, moderate and high (1–1,000 pollen/m^3^ for trees and 1–100 pollen/m^3^ for grass and Chrysanthemum Weed) during the investigation period of the PA group. No days with very high pollen content occurred during the exposure period (Personal communication from Åslög Dahl, Department of Plant and Environmental Sciences, Gothenburg).

No differences were found concerning age and smoking habits between the groups. There was also no difference between the two groups of hairdressers with regard to employment years as a hairdresser, working hours or atopy by skin prick test (Table 1).

**Table 1:** Characteristics of the symptomatic (S+) and asymptomatic hairdressers (S−) and pollen allergic women (PA)

Study groups	S+ *n* = 17	S− *n* = 19	PA *n* = 10
Age (years; mean; SD)	39 (11)	37 (12)	34 (15)
Employment years as a hairdresser (mean; SD)	20 (13)	17 (12)	–
Working activity as a hairdresser (*n*)			
<50 %	3	2	–
51–75 %	6	6	–
76–100 %	8	11	–
Smoking habits (*n*)			
Smokers	2	2	0
Never smokers	13	17	9
Ex smokers	2	0	1
Atopy–by history test (*n*)	0	0	10
Positive skin prick test (*n*)	1	2	10

### Clinical examination

A physician (JN) conducted a standardized interview including a medical and occupational history, questions about atopy and smoking habits. Special attention was given to airway-related symptoms and their relationship to the workplace. Work-related rhinitis was defined according to the position paper for occupational rhinitis by Moscato et al. (2008) and by Sublett and Bernstein (2010). Atopy by history was defined as having a history of hay fever, asthma or atopic eczema in childhood or adolescence.

A physical examination was performed including an anterior rhinoscopy and a skin prick test with 13 common allergens (ALK, Copenhagen, Denmark) and potassium persulphate in fresh solutions with sterile water [0.05, 0.1 and 0.5 % (w/v)]. The reaction was read according to Aas and Belin (1973). The medical examination for the atopics including the quality of life questionnaires took place before the start of the pollen season.

### Diary

During 4 weeks of exposure, all study subjects filled in a diary including symptoms from the eyes, nose, throat, cough, sputum production, wheezes, dyspnea, cold/flu symptoms, medication use and if they had been staying out of work due to their symptoms. The hairdressers also stated what hair treatments they accomplished daily, such as bleaching, hair dyeing, hair spraying, applying permanent and the type of products used. They indicated use of ventilation and other protective products such as gloves and apron. The PA group started the diary when having clear allergic symptoms and documented if they reacted to any other agent than pollen. In the results section, symptoms caused by infection are excluded.

### Nasal lavage

A nasal lavage was performed before the exposure period for all subjects. Repeat nasal lavage was performed after 1 week and again after 4 weeks of exposure for the hairdressers. Allergic subjects had repeat nasal lavage only after 4 weeks. The lavage was performed using sterile isotonic saline solution. This was sprayed into the nasal cavity using a container of glass and a plastic atomizer nozzle. A centrifuge tube was placed in crushed ice and topped with a plastic funnel. The saline was sprayed three times into each nostril at the nasal conchae. The study subject was instructed to breathe by the mouth and to lean forward and let the fluid drop from the nostrils into the funnel until 10 mL was collected in the tube. The tubes were kept on ice until centrifugation, which was performed within 3 h (Naclerio et al. 1983; Quirce et al. 2010).

### Analysis of the nasal lavage

The supernatant was obtained by centrifugation of the sample volume at 0.3 g for 10 min at 4 °C. The samples were kept at −80 °C until analysis. For Substance P, one ml of nasal lavage fluid was transferred into a 3.6 mL Nunc cryotube containing 1 mL of inhibitor. For ECP and tryptase analysis, the supernatant was transferred into a 3.6-mL cryotube. We could not exclude blood in the nasal lavage samples, and therefore, we did not include the data for albumin.

The levels of ECP and tryptase were analyzed by a double antibody fluoro enzyme immunoassay. These assays are available as commercial kits (Pharmacia Diagnostics AB, Uppsala, Sweden). Substance P was analyzed by an Immuno Linked Immuno Assay, ELISA (Cayman Chemical Company, Ann Arbor, MI, USA). The detection limit for albumin was 0.4 mg/L, for ECP 0.5 μg/L, for Substance P 8.2 ng/L and for tryptase 1.0 μg/L.

### Specific nasal challenge

A specific nasal challenge was performed before and after 4 weeks of exposure in the S+ group. The challenge was made with a 0.001 % fresh solution of potassium persulphate in isotonic saline solution and after 20 min with a 0.01 % solution (w/v) using a de Vilbiss spray (atomizer No. 15) as earlier described (Nielsen et al. 1994). A total of 300 μg of each solution was sprayed into the nasal cavity by turns. The spraying was performed immediately after a maximal inspiration to prevent the solution from entering the lower airways (Mellilo et al. 1997).

Nasal symptoms (blockage, running nose) were recorded using a score system from 0–3 (0 = no symptoms, 3 = severe symptoms) before and 15 min after each challenge. The rating was performed for each nostril, and the average was used. The number of sneezes was counted and scored as “no sneeze attacks” = 0; 1–5 = 1; 6–15 = 2; >15 = 3. A combined nasal symptom score was calculated from nasal blocking, secretions and sneezes (Malm et al. 1981).

Acoustic rhinometry (AR) was performed using a RhinoScan v. 2.5 (Interacoustics A/S, Assens, Denmark) according to Hilberg and Pedersen (2000). The measurements were made as earlier described in Kronholm Diab et al. (2009). The Minimum Crossectional Area and the volume were measured in the distance of 10–22 mm (MCA1 and VOL1) and 22–54 mm (MCA2 and VOL2) from the nares before the challenge and 15 min after each challenge. Data are presented as the mean of the values for the right and the left side of the nose. Nasal reactivity was defined as a significant increase in nasal symptoms of ≥3 points in total symptom score (Kronholm Diab et al. 2009) and/or a significant decrease in AR measures of the anterior part of the nasal cavity (Hilberg and Pedersen 2000).

A nasal lavage was performed twice before the first challenge and 20 min after the second one directly after the rhinometric measurement. The first lavage (Time 0) was performed to wash out mediators due to the general environmental exposure before the challenge. The second lavage (Baseline) before the challenge was used as the baseline for the post-challenge samples. The lavage procedure was made as earlier described in Kronholm Diab et al. (2009).

### Quality of life questionnaires

The study participants filled in the Short Form 36 Health Survey (SF-36) (Ware and Sherbourne 1992; Ware et al. 1993) and the Rhinoconjunctivitis Quality of Life Questionnaire (RQLQ) (Juniper and Guyatt 1991; Juniper et al. 1996) before the medical examination to avoid influence from the questions posed by the physician. The participants were instructed according to the guidelines defined by the designers of the questionnaires. As proposed by several authors, we used a combination of generic and disease specific quality of life scales (Leong et al. 2005; Terreehorst et al. 2004). The study participants were asked if any serious or dramatic event had happened during the observation period to exclude response shift (van Gerth Wijk 2002). In the comparison analyses for quality of life, the number of participants in the S− group is 18, due to missing questionnaires from one hairdresser at the study end.

#### SF-36

The SF-36 was given to analyze the hairdressers last 4 weeks. We used the Swedish self-administered version (Sullivan et al. 2002). SF-36 comprises 36 items within eight health domains related to physical and mental health dimensions: PF (Physical Functioning, 10 questions), RP (Role Physical Functioning, 4 questions), BP (Bodily Pain, 2 questions), GH (General Health, 5 questions), VT (Vitality, 4 questions), SF (Social Functioning, 2 questions), RE (Role Emotional, 3 questions) and MH (Mental Health, 5 questions). The domains are scored on a scale of 0 (worst) to 100 (best) points and calculated for each domain using a standardized scoring system (Sullivan et al. 2002; Ware et al. 1993). On the basis of the eight scales, it is also possible to estimate a physical (PCS) and a mental component summary (MCS) score (Ware et al. 1995). The Swedish version of the SF-36 has shown good psychometric values in different studies (Taft et al. 2004), and there are norms for the Swedish population available (Sullivan and Karlsson 1998).

#### RQLQ

Rhinoconjunctivitis quality of life questionnaire (RQLQ) evaluates QoL in a particular disease state (Juniper and Guyatt 1991). This 28-item questionnaire is self-administered and has been validated to measure the functional impact of rhinoconjunctivitis in seven domains: activity limitation, sleep, Non-rhinitis symptoms, practical problems, nose and eye symptoms and emotional function. The domains are scored from 0 (=no impairment) to 6 (=severe impairment) as perceived by the subject during the previous week. The RQLQ has strong evaluative and discriminatory properties (Juniper et al. 2002).

### Statistical analysis

For all statistical analyses, SPSS version 15.0 and PASW 18.0 (SPSS Inc., Chicago, IL, USA) were used.

The eight health indices in SF-36 were calculated according to a SAS program provided by the HRQL group at the Sahlgrenska University hospital in Gothenburg (www.hrql.se), who handles the Swedish version of SF-36. We calculated mean, standard deviation (SD) and 95 % confidence interval as parameters for the QoL data, as the SAS program delivers mean values and SD. Visually assessed p–p-plots suggested that the data were normally distributed. For comparisons between groups, the Mann–Whitney U test was employed, and for changes within the groups, the Wilcoxon signed-ranks test. This is also valid for the analysis of biomarkers and symptoms. The significance level was set at 5 %.

Variables with dichotomous outcomes were analyzed with a generalized model with a logit link (i.e., logistic regression). Continuous variables were analyzed with a linear mixed model with restricted maximum likelihood (REML) estimation and a diagonal covariance matrix. In both models, repeated measures were identified by personal identification number and day in study. For the continuous variables “High-lifting blond,” “Hair Dye,” “Blond Hair Dye” and “Brown Hair Dye,” the final Hessian matrix was not positive. These were therefore dichotomized into the categories 0 and ≥1 and analyzed with the logit link.

## Results

### Diary

#### Symptoms and medication used

**Fig. 2:**
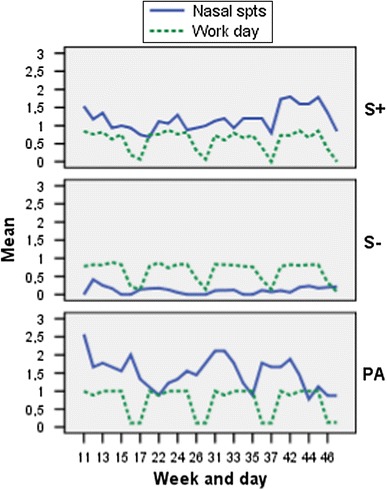
Nasal symptoms (blockage, itching, sneezing, secretion; Mean) without infection and work days in symptomatic (S+; *n* = 17) and asymptomatic (S−; *n* = 19) hairdressers and pollen allergic women (PA; *n* = 10)

The S+ group had increased nasal symptoms steadily during the exposure period. The PA group had more nasal symptoms (running, itching nose, sneezes) from the start than the S+ group, and the symptoms varied from week to week (Table 2). The eye symptoms varied less than the nasal symptoms. The OR for eye symptoms in the PA group compared to the S+ group was 8.07 (CI 95 % −3.20, −0.98; *P* < 0.001). In relation to the working days, the number of symptoms in the S+ group decreased during weekends and had a clear increase during the work days, especially at the end of the study period contrary to the PA group whose symptoms increased during days off work (Fig. 2). When the different nasal symptoms were studied separately, the S+ group had less sneezing and a tendency to more blockage than the PA group (Table 3). Nasal decongestants were consumed in the S+ group only during two percent of the study days. The PA group took antihistamines during 30 % of the study days. Furthermore, 8.2 % of the days they took antihistamines in combination with other allergy medications (data not shown). No significant differences were seen between the symptomatic hairdressers and the pollen allergic women regarding throat irritation, hacking cough, sputum or wheeze. However, the symptomatic hairdressers had more throat irritation (OR 1.13, CI 95 % −1.12, 1.37; ns) than the pollen allergic women (data not shown).

**Table 2:** Total nasal symptoms per week during the observation period (median; range) in symptomatic (S+) and asymptomatic (S−) hairdressers and pollen allergic women (PA)

Study groups	S+ *n* = 17	S− *n* = 19	PA *n* = 10	*P* values		
				S+ ↔ S−	S+ ↔ PA	S− ↔ PA
Week 1	7 (0–18)	0 (0–9)	14 (0–20)	0.001	0.011	<0.001
Week 2	8 (0–16)	0 (0–7)	8.5 (0–21)	<0.001	ns	<0.001
Week 3	8 (0–18)	0 (0–3)	15.5 (0–22)	0.001	ns	0.001
Week 4	11 (0–25)	0 (0–14)	7.5 (0–19)	<0.001	ns	0.001

Blocking, secretion, itching, sneezing. Symptoms caused by present infection are excluded

*ns* non-significant

**Table 3:** OR, CI 95 % and *P* values for nasal symptoms in the symptomatic (S+) and the asymptomatic hairdressers (S−) compared to the pollen allergic women (PA) during the observation period

Nasal symptoms	S+ *n* = 17		S− *n* = 19		*P* value	
	OR	CI 95 %	OR	CI 95 %	S+	S−
Blockage	1.23	(0.41–3.70)	0.04	(0.01–0.15)	ns	<0.001
Itching	0.69	(0.26–1.85)	0.05	(0.01–0.33)	ns	<0.001
Sneezing	0.30	(0.12–0.74)	0.06	(0.02–0.25)	0.010	<0.001
Secretion	0.52	(0.18–1.52)	0.02	(0.0–0.06)	ns	<0.001

#### Exposure

Although the S+ group had a tendency to perform more hair treatments such as bleach, high-lifting blond and hair dye than the S− group, the only significant difference was in the use of hair spray (Mean S+ 3.0, S− 2.3; Mean difference −0.569, CI 95 % −0.917 to −0.221; *P* = 0.001). Within the S+ group, there was a tendency to less numbers of hair treatments during the last part of the study period (data not shown). There were no significant differences in the type of bleaching powder used such as dust, granules and crème, nor the type of hairspray (pump or aerosol propellant). Local exhaust ventilation was infrequently used in both groups (data not shown).

### Nasal lavage and specific nasal challenge

#### Inflammatory markers

The S+ group increased in ECP during the study period, and the S− group did not. The PA group had a higher level ECP, but no significant increase during the study period was noticed (Table 4). No significant differences regarding Substance P and Tryptase were registered between the S+ and S− groups during the study period. There was no significant difference in tryptase levels before and after the study period in the PA group (data not shown).

**Table 4:** Eosinophil cationic protein (ECP, µg/L) in nasal lavage fluid in symptomatic (S+) and asymptomatic (S−) hairdressers and pollen allergic women (PA) before (BE), after 1 (1wAE) and 4 weeks (4wE) of exposure

ECP (µg/L)	S+ *n* = 17		S− *n* = 19		PA *n* = 10		*P* value	
	Median	CI 95 %	Median	CI 95 %	Median	CI 95 %	S+↔ S−	S− ↔ PA
BE	3.8	(1.9–149.0)	4.0	(1.9–22.0)	8.4	(3.7–41.0)	0.523	0.002
1wAE	5.9	(1.9–57.0)	3.7	(1.9–32.0)	–		0.273	
4wAE	7.0^a^	(1.9–141.0)	3.1	(1.9–11.0)	28.0	(1.9–200.0)	0.050	0.002

^a^1wAE ↔ 4wAE *P* = 0.050

#### Specific nasal challenge

At the specific nasal challenge in the S+ group, the total nasal symptom score before challenge increased from 1 before work started to 2 after 4 weeks (Median; *P* = 0.022). After the first challenge, the symptom score increased from 1 to 2 (*P* = 0.005) and after 4 weeks of exposure the score increased from 2 to 3 (*P* = 0.006) indicating no change in nasal reactivity. The sub-group of those who reacted significantly at the first challenge did not react more at the second challenge compared to the non-reactors. No significant changes were found in acoustic rhinometry (data not shown). Before work started, albumin increased significantly from baseline to after the second challenge, while after 4 weeks of work the same increase was not significant (Table 5).

**Table 5:** Albumin (mg/L) and Substance P (µg/L) (median; range) in nasal lavage fluid at specific challenge with per sulphate in symptomatic hairdressers (*n* = 17) after vacation and after four weeks of exposure

	BE		AE	
Albumin (mg/L)				
Time 0	4.2	(0.3–57.0)	4.7	(0.6–22.0)
Baseline	2.0	(0.6–17.0)	2.4	(0.3–14.0)
20 min after challenge 2	4.0^a^	(0.5–19.0)	3.7	(0.3–11.0)
Substance P (µg/L)				
Time 0	9.5	(4.3–44.4)	12.2	(6.4–34.8)
Baseline	8.9	(0.0–29.3)	12.6	(4.2–33.0)
20 min after challenge 2	10.9^b^	(3.9–60.7)	12.1	(3.9–40.6)

*BE* before and *AE* after four weeks of exposure

*P* value: ^a^ 0.047 baseline ↔ after challenge 2, ^b^ 0.030 baseline ↔ after challenge 2

### Health-related quality of life

#### Summary indexes

Before the exposure, the S+ and the PA groups had approximately the same Overall QoL. The S− had a better score compared to the other two groups (Table 6). After the study period, the hairdresser groups did not change significantly, whereas the PA group was significantly worse with a mean difference of 0.8. In the SF 36 before the study, the two hairdresser groups did not differ and had a higher score than the PA group in the mental summary score, though not significantly. No significant changes were noticed within the groups after the observation period (data not shown). During the exposure period, two S+ and one S− hairdressers as well as one participant from the PA group had experienced personal problems. Two S+ hairdressers had developed eczema to hairdresser chemicals. These events did not influence the results of the questionnaires, which we tested for by analyzing and comparing the data including and excluding these persons.

**Table 6:** Selected domains from the RQLQ and the SF-36 before (BE) and after (AE) the study period in symptomatic (S+) and asymptomatic (S−) hairdressers and pollen allergic women (PA)

Measures	Health domains	Groups	BE		AE		Changes BE to AE		
			Mean	SD	Mean	SD	Mean	SD	*P* value
*Overall QOL*									
RQLQ^1^	Overall QoL (Mean of all 7 domains)	S+	1.2^c^	1.4	1.5^aaa^	0.9	0.4	1.5	0.327
		S−	0.2	0.3	0.3	0.7	0.1	0.8	0.594
		PA	1.2^ccc^	1.2	2.0^ccc^	1.5	0.8	0.8	0.014
*Physical domains*									
RQLQ^1^	Non-hay fever spt	S+	1.2^a^	1.5	1.7^aaa^	1.0	0.6	1.7	0.183
		S−	0.8	0.2	0.2	0.8	1.6	0.9	0.449
		PA	1.3^cc^	1.4	2.1^cc^	1.4	0.8	0.9	0.021
	Nasal spt	S+	1.2^aa^	1.5	1.7^aaa^	1.0	0.6	1.9	0.221
		S−	0.2	0.5	0.5	1.0	0.3	1.1	0.211
		PA	1.2^ccc^	1.3	2.0^cc^	1.4	0.8	1.0	0.031
	Eye spt	S+	1.0^aa^	1.0	1.2^aaab^	1.4	0.2	1.5	0.508
		S−	0.2	0.5	0.0	0.1	1.6	4.2	0.119
		PA	0.9^cc^	1.2	2.3^cc^	1.4	1.5	1.2	0.005
SF-36^2^	Physical Functioning (91;16)^3^	S+	95.0^a^	9.0	94.1^a^	14.4	0.9	4.8	0.693
		S−	97.2^b^	4.3	96.3^b^	6.0	1.1	4.8	0.435
		PA	84.9	14.3	84.4	11.8	0.4	11.4	0.912
	Role–Physical (86;30)^3^	S+	88.2	28.1	70.6	39.8	17.6	41.2	0.097
		S−	85.2	30.2	97.2	11.8	12.0	29.0	0.097
		PA	86.1	22.0	77.8^cc^	34.1	8.3	28.0	0.397
*Mental domains*									
RQLQ^1^	Activities	S+	1.5^aa^	1.7	1.8	1.1	0.3	1.7	0.437
		S−	0.3	0.2	0.2	0.8	0.1	1.0	0.523
		PA	1.5^c^	1.4	2.8	2.0	1.3	1.7	0.036
SF-36^2^	Vitality (67;2)^3^	S+	70.3	18.4	59.4^aa^	20.5	10.9	20.6	0.044
		S−	73.1	16.2	77.8	15.6	4.7	12.7	0.132
		PA	59.4	28.8	52.2^c^	29.3	7.2	11.5	0.096

^1^Higher score means worse QOL

^2^Higher score means better QOL

^3^Numbers in brackets are the Swedish norms for Females aged 30–49; *n* = 1731

S+ versus S−; ^a^ *P* ≤ 0.050, ^aa^ * P* ≤ 0.010, ^aaa^ *P* ≤ 0.001

S+ versus PA; ^b^ *P* ≤ 0.050, ^bb^ * P* ≤ 0.010, ^bbb^ *P* ≤ 0.001

S− versus PA; ^c^ *P* ≤ 0.050, ^cc ^ *P* ≤ 0.010, ^ccc^ *P* ≤ 0.001

#### Physical domains

##### RQLQ

Before the exposure period, the S+ and the PA groups were at the same level in the RQLQ physical items. The S− had better scores than the other two groups (Table 5). The most notable change during the study period in the S+ group was a slight deterioration in Nasal and Non-hay fever symptoms. The S− tended to become better after the work weeks except for in the category of nasal symptoms. The PA group had significant deteriorations in Eye, Nasal and Non-rhinitis symptoms (Table 6). There was a significant difference between the S+ and the PA groups in Eye symptoms after the exposure, and between the PA and the S− groups before and after in all three items (*P* < 0.050).

##### SF-36

The two hairdresser groups had significantly better scores than the PA group in the Physical Functioning before as well as after the study period (Table 6). For the other domains, there were no significant differences between the groups before the study. After the study period, the S+ and the PA group tended to decrease and the S− group increased in Role Physical. Thus, significant differences were found between the S−, S+ and PA, respectively (Table 6). No significant changes were noticed within the groups from before exposure to after (data not shown).

#### Mental domains

##### RQLQ

The S+ and the PA groups were at the same levels before the exposure, while the S− had a better quality of life within the mental items (Table 6). There were significant differences between the S groups in Activities, Practical problems, and Emotions before and after the observation period, and also in Sleep after the exposure. Between the S− and the PA groups, there were significant differences in all items both before and after the work weeks (*P* ≤ 0.050). No significant changes were noticed within the groups during the study period except for the PA group who showed a significant deterioration in Activities (Table 6).

##### SF-36

Before the work period, the two S groups had about the same scores in the mental health domains, whereas the PA group tended to have a lower score (Table 6). After the work period, the S+ and the PA groups showed a decrease and the S− group an increase in Vitality. Thus, significant differences were found between the S− and the S+ and the PA groups, respectively. The mean difference for Vitality in the S+ group after the study period was 10.9, while no significant differences were seen in the other groups.

## Discussion

In this study, we wanted to take a comprehensive look at the physical and psychological impact of chemical exposures hairdressers have at work. The hairdressers’ nasal symptoms, mainly nasal blockage, increased steadily during the observation period, although they improved during weekends. There was an increase in ECP in nasal lavage fluid but the nasal reactivity to persulphate did not increase. The HRQoL deteriorated in the physical as well as in the mental domains in the symptomatic hairdressers especially in Vitality (SF-36). Notably, the asymptomatic hairdressers tended to ameliorate their HRQoL during work, while the pollen allergic group was more impacted than both hairdresser groups.

### Methodology

The participants in the S+ group were recruited from current patients at the clinic fulfilling the inclusion criteria. As very few refused to participate, we think that a selection bias is less likely. Furthermore, our groups were rather small; thus, we may miss some weak correlations. Our study period was also short. However, the risk of missing data would have increased as the loss of participants in prospective studies is a well-known problem (Kristman et al. 2004). In our case, the hairdressers used to have frequent short vacancies; thus, longer observation periods with exposure was not possible. Another reason we chose a relatively short study period was to ensure compliance with journaling among participants.

The hairdressers were compared to a group of pollen allergic women. It was not practically possible to define a zero point with regard to exposure for the PA group in the same way as for the hairdressers. This affected the results in the study of the mediators and the symptoms at the start of the diary.

We examined the HRQoL by choosing the SF-36 questionnaire, an extensively used generic quality of life questionnaire with acceptable discriminative but poorer evaluative properties for measuring rhinoconjunctivitis specific quality of life, and the RQLQ, which has strong discriminative and evaluative properties (Juniper et al. 2002). Specific questionnaires seem to be more sensitive to changes in HRQoL over time. However, the SF-36 has shown to be a complement of good value to the disease specific RQLQ (Leong et al. 2005; Terreehorst et al. 2004). The results of SF-36 are compared to the Swedish norms (Sullivan and Karlsson 1998). However, these are from 1991–1992 and may not be fully relevant due to changes in the society. Thus, our comparisons to these norms should not be over interpreted.

### Diary and inflammatory markers

The clinical picture differed between the symptomatic hairdressers and the pollen allergic women. The hairdressers reported less symptoms from the eyes and more nasal blockage than the atopics, who had more itching, sneezing and secretion. The mechanism of the hairdressers’ symptoms is not clear. The meaning of specific IgE against persulphates in the mechanism of hairdressers’ nasal symptoms and also the use of skin prick testing in the diagnostics are controversial. We did not in an earlier study (Kronholm Diab et al. 2009) find specific antibodies using immunoblotting, and neither did we find any positive skin prick tests in that study, nor in the present one. Thus, the hairdressers’ nasal symptoms may not be elicited through an IgE-mediated reaction to persulphates contrary to the symptoms in the pollen allergic group. Of course, IgE-mediated reactions could be elicited by other agents in the hairdressers salons, and in fact Hollund et al. (2002) found increased levels of total IgE in highly exposed hairdressers, but not after adjustment for age, atopy and smoking. Sensitization to latex was found by Hollund et al. (2002) and Leino et al. (1998) in some hairdressers, but the latter concluded that sensitization to agents other than persulphates is not common among hairdressers. The present hairdressers did not use latex gloves. Furthermore, in another study of nasal symptoms associated with exposure to organic acid anhydrides, those subjects who were not IgE sensitized to the anhydrides complained of nasal congestion and the sensitized ones of nasal secretion and sneezing (Nielsen et al. 2006). Thus, the difference in the clinical picture in hairdressers and in pollen allergic women may be due to different mechanisms.

The group of symptomatic hairdressers showed a slight but stable increase in nasal symptoms during the study period with transient decreases during days off. Furthermore, the increase in ECP during the study period indicated a progressive effect on the nasal mucosa from exposure. In the pollen allergic group, the symptoms varied during the observation period probably due to the level of exposure but the ECP level in nasal lavage increased. The reactivity to potassium persulphate in the nasal challenge test did not increase during the observation period in the symptomatic hairdressers all together. Looking at the sub-groups of those having an increase in nasal symptoms at the first challenge or not, neither of the sub-groups had a significant increase in nasal symptoms at the challenge after 4 weeks of work. Thus, it may be due to a too short observation period or that other agents than the persulphates may be the cause of the symptoms (Mounier-Geyssant et al. 2006; Ronda et al. 2009). It is possible that the reactions in the symptomatic group may simply be due to a higher level of exposure to chemicals and not to a sensitization to one or more chemicals. Opposed to this view, the hairdressers had a tendency to decrease the number of treatments during the study period. Furthermore, in an earlier study by our group, we have shown that there is a clear difference in reactivity between symptomatic and asymptomatic hairdressers when challenged with potassium persulphate indicating some form of sensitization (Kronholm Diab et al. 2009). Therefore, the mechanism behind the hairdresser’s symptoms needs to be further examined.

### Health-related quality of life

The results of this study indicated a better HRQoL in the two groups of hairdressers at study start compared to the Swedish female references for SF-36 except for General Health in the symptomatic hairdressers. The symptomatic hairdressers had a somewhat lower HRQoL than the asymptomatic ones. Two earlier studies have shown that the HRQoL among patients no longer exposed improves (van Gerth Wijk et al. 2011) or becomes similar to that of healthy controls (Airaksinen et al. 2009). In the present study, the symptomatic hairdressers may have had a too short time off for a total recovery, which is also supported by the fact that they still had nasal symptoms at the study start.

Before the study period, the pollen allergic women had a decreased Vitality, an important aspect of the General Health showing how strong or weak, energetic or tired and worn out one feels, compared both to the hairdresser groups and to the Swedish norms. The same was true regarding Physical Functioning pointing out limits in the function of physical activities. The reason the pollen allergic group had a lower HRQoL than the hairdressers before the study period is not clear. They were either working or studying; thus, there should not be any healthy worker effect. It may be an effect of a chronic disease in the atopics, may represent the hairdressers’ overall job satisfaction or simply an effect of the hairdressers having at least 2 weeks off work at study start, which the atopics did not (Riise and Moen 2003).

The asymptomatic hairdressers had an improvement in their HRQoL during the study period contrary to the symptomatic group who deteriorated parallel to the increase in symptoms. The symptomatic group finished the study period with the same inferior level as bell pepper greenhouse workers with rhinitis related to allergen exposure (Groenewoud et al. 2006). The pollen allergic women decreased significantly during the study period in both physical and mental domains in accordance with earlier studies (Camelo-Nunes and Sole 2010; Valovirta et al. 2008).

Juniper et al. (1996) have provided evidence for the minimal important difference (MID) to be 0.5 in RQLQ indicating that changes in the score of ≥0.5 can be considered of clinical importance. The symptomatic hairdressers showed a MID ≥ 0.5 in Non-rhinitis symptoms (lack of energy, thirst, reduced performance capacity, tiredness, concentration difficulties, headache, feeling of worn out) and in Nasal symptoms indicating most clinical effects in these domains. The deterioration in Non-rhinitis symptoms conforms well to the decrease in Vitality in the SF-36, thus the two results supporting each other. This strengthens our conclusion that there was a negative effect on the HRQoL of the symptomatic hairdressers during work.

In conclusion, the difference in the clinical picture between the symptomatic hairdressers and the pollen allergic females, and the increasing rates of symptoms and inflammation markers in the nasal mucous membrane during the study period support the view that a sensitization to hairdresser chemicals by a mechanism not yet understood is operating. Although the symptomatic hairdressers had a better HRQoL than the atopics before the study period/season, they had a considerable deterioration during exposure contrary to the asymptomatic hairdressers.
